# 10th Annual Conference on New and Re-emerging Infectious Diseases

**DOI:** 10.3201/eid1311.070791

**Published:** 2007-11

**Authors:** Uriel Kitron, Brenda A. Wilson

**Affiliations:** * University of Illinois at Urbana-Champaign, Urbana, Illinois, USA

**Keywords:** emerging, conference, re-emerging, conference summary

The 10th Annual Conference on New and Re-emerging Infectious Diseases, held in Urbana, Illinois, USA, April 19–20, 2007, featured 6 speakers and >30 posters. This year the conference was sponsored by the Conservation Medicine Center of Chicago and by the following units of the University of Illinois at Urbana-Champaign: the Center for Zoonoses Research; the Department of Pathobiology; the Environmental Council; the Host-Microbe Systems Theme of the Institute for Genomic Biology; and the Program in Arms Control, Disarmament, and International Security.

Marta Guerra, from the Centers for Disease Control and Prevention (CDC), a graduate of the University of Illinois, opened the conference with a presentation on epidemiologic investigations and responses at CDC. As an Epidemic Intelligence Service officer and a CDC public health officer in Uganda, Ethiopia, and the United States, she dealt with Ebola virus, raccoon and skunk rabies, monkeypox, Q fever, severe acute respiratory syndrome, and the global polio eradication campaign. Her talk was of particular interest to those veterinary students who are interested in pursuing a career in public or global health.

Doug Goodin, Kansas State University, presented a framework for the ecology of infectious disease based on consideration of landscape hierarchy and spatial scale in the study of hantavirus dynamics in Paraguay. The fundamental concept of hierarchy theory is that processes occurring at finer scales (i.e., “lower” in the spatial hierarchy) are constrained by processes that occur at higher levels. Hierarchical levels can also be distinguished by the rates at which processes occur—faster at finer scales, slower at coarser ones. Such a framework is amenable to the study of landscape epidemiology, because the linkages between environmental factors and disease are neither univariant nor confined to a specific spatial scale.

Uriel Kitron, University of Illinois at Urbana-Champaign, discussed environment, change, and disease illustrated by the interactions of several vectorborne zoonoses in the Americas with urbanization, agricultural development, forestation, and global warming. In particular, on the basis of findings from 2 studies supported by the National Science Foundation and the National Institutes of Health Ecology of Infectious Disease program, West Nile virus showed the introduction, establishment, and distribution of a new zoonosis in an urban area, and Chagas disease showed the persistence of a zoonosis, despite changes in vector distribution, habitat modification, and the role of various zoonotic hosts in the Gran Chaco of Argentina.

Helen Jost, University of Arizona, described the unusual toxins of *Arcanobacterium hemolyticium*, an emerging pathogen and an important cause of bacterial pharyngitis in adolescents and young adults. *A. hemolyticum* expresses an unusual phospholipase D (PLD) with amino acid similarity to recluse spider venom. The addition of a cloned and purified recombinant PLD to HeLa cells resulted in substantial remodeling of the host membrane architecture, as measured by lipid raft formation. She also described a newly identified member of the cholesterol-dependent cytolysin family, arcanolysin (ALN), expressed by *A. hemolyticum*. Both PLD and ALN are membrane-active toxins and contribute to the adhesion and invasion of *A. hemolyticum* to host cells. Therefore, these molecules may contribute to the pathophysiologic processes of invasive *A. hemolyticum* infections.

J. Stephen Dumler, Johns Hopkins University, discussed a novel pathogen and pathogenic mechanisms of *Anaplasma phagocytophilum*, a tick-transmitted obligate intracellular bacterium that has emerged to become the third most common vectorborne infection in North America. In vitro studies have shown that neutrophil infection by *A. phagocytophilum* induces notable functional changes, many based on altered transcription in the host. A novel protein call AnkA in *A. phagocytophilum* is translocated from the bacterium within a host vacuole into the host nucleus, where it forms complexes with heterochromatin and is largely responsible for many host transcriptional changes by directly binding to regulatory regions of the DNA. This binding leads to altered eukaryotic histone structure and the potential recruitment of some transcriptional activators or repressors to multiple loci in the myeloid cell chromosomes. The net effect is continued inflammatory recruitment of new hosts and lack of microbicidal mechanisms, rendering a higher proportion and prolonged presence of pathogen accessible to tick bites.

Tamara Maier, Medical College of Wisconsin at Milwaukee, presented an analysis of *Francisella tularensis* Himar1-based transposon mutants that are defective for replication in macrophages. She and her colleagues have developed genetic approaches to screen for virulence factors as potential targets for therapeutic or vaccine development. A Himar1-based transposon system was constructed, optimized, and used to create mutants in the *F. tularensis* strain LVS. Based on a simple cell retention assay, a library of ≈7,000 insertion mutants was screened in J774A.1 macrophages for a reduction in the cytopathic effects. Genes that were identified through their screens were involved in a variety of processes, including transport, metabolism, and cell wall and membrane biogenesis, which could potentially encode required gene products for the replication of *Francisella* spp. in murine macrophages.

The final speaker was Roberto DoCampo, University of Georgia, a founder of the conference. He discussed novel targets for treatment of American and African trypanosomiases. His laboratory has been investigating 2 organelles (the acidocalcisome and the contractile vacuole) and 1 metabolic pathway (the isoprenoid pathway) for target identification. Ablation by RNA-mediated interference (RNAi) of several acidocalcisome proteins of *Trypanosoma brucei* has demonstrated that acidocalcisomes play an essential role in osmoregulation and intracellular pH regulation. The enzyme farnesyl diphosphate synthase (FPPS) of *Trypanosoma cruzi* was cloned and sequenced, and the genes encoding FPPS were expressed in *T. brucei*. The protein products are inhibited by bisphosphonates, and the 3-dimensional structure is bound to different bisphosphonates. A newly identified solanesyl diphosphate synthase, which is involved in the synthesis of the solanesyl group, is necessary for the synthesis of ubiquinone. This enzyme is also potently inhibited by bisphosphonates and represents a novel target for the treatment of trypanosomiasis. Proceedings are available in PDF format from www.cvm.uiuc.edu/idc

